# Nitric oxide is negatively correlated to pain during acute inflammation

**DOI:** 10.1186/1744-8069-6-55

**Published:** 2010-09-15

**Authors:** May Hamza, Xiao-Min Wang, Tongtong Wu, Jaime S Brahim, Janet S Rowan, Raymond A Dionne

**Affiliations:** 1 NINR/NIH, (10 Center Drive), Bethesda, MD (20892), USA; 2 Department of Epidemiology and Biostatistics, University of Maryland, 1242C HHP Building, Collage Park, MD (20742), USA; 3 Department of Oral-Maxillofacial Surgery, University of Maryland, Baltimore Dental School, Baltimore, MD USA; 4 Department of Nursing, Magnuson Clinical Research Center, NIH, Bethesda, MD (20892), USA; 5 Department of Pharmacology, Faculty of Medicine, Ain Shams University, Abbassia, Cairo (11566), Egypt

## Abstract

**Background:**

The role that nitric oxide (NO) plays in modulating pain in the periphery is unclear. We show here, the results of two independent clinical studies (microdialysis and gene expression studies) and a pilot dose finding study (glyceryl trinitrate study), to study the role of NO in the early phase of acute inflammatory pain following oral surgery. The effect of ketorolac on NO production and nitric oxide synthase (NOS) gene expression was also studied.

**Results:**

Microdialysis samples showed significantly higher levels of NO at the first 100 min compared to the last 80 minutes in the placebo treated group. In the ketorolac group, on the other hand, NO levels gradually decreased over the first 60 min but were similar to placebo over the later 100-180 min, with no significant change in NO level over time. The levels of NO were negatively correlated to pain intensity scores. Local infusion of the NO donor glyceryl trinitrate at the site of surgery, showed a small analgesic effect that did not reach statistical significance in the sample size used. While the gene expression of iNOS and eNOS were not up-regulated, 3 hours after surgery, nNOS was downregulated in both treatment groups and eNOS gene expression was significantly lower in the ketorolac group compared to the placebo group. Further, there was a positive correlation between the change in gene expression of nNOS and eNOS in the placebo goup but not in the ketorolac group.

**Conclusion:**

We suggest that at this early stage of inflammatory pain in man, NO is analgesic in the periphery. Further, ketorolac down-regulates eNOS gene expression.

## Background

Nitric oxide (NO) is involved in numerous physiological processes in the peripheral and central nervous system [1]. It is produced intracellularly by the catabolism of L-arginine to L-citrulline by NO synthase enzyme (NOS), which is present in three isoforms, the neuronal (nNOS), endothelial (eNOS) and inducible (iNOS) isoforms. The role that NO plays in pain is not simple, since it may show pro- or anti-nociceptive effects depending on the circumstances [2]. The majority of data, in preclinical studies support a pronociceptive role of NO at the spinal level [3-6]. Yet, other studies show inconsistent results; for review see [7].

The effect of NO on pain and analgesia in the periphery is further conflicting. Sodium nitroprusside, which releases NO non-enzymatically, has an antihyperalgesic effect in the rat paw pressure test [8]. Conversely, intradermal administration of the NOS substrate L-Arg or the NO donor SIN-1, both of which elevate NO levels, cause a dose-dependent mechanical hyperalgesia [9]. Conflicting clinical effects are also reported; as intracutaneous injection of NO in healthy volunteers evokes pain in a dose dependent manner [10], while transdermal application of the NO donor glyceryl trinitrate improved pain in patients with shoulder pain syndrome [11]. Associations between NO levels and pain intensity also show conflicting results. While there was no association between NO concentration in the perifacetal region and pain duration or pain level in patients with chronic low back pain [12], NO correlated with pain in the polyarticular subtype of juvenile idiopathic arthritis with active disease[13].

The role of different NOS isoforms in inflammatory pain process is derived primarily from animal experiments [2]. While nNOS is mainly observed at the spinal level or in neuropathic pain models, iNOS is up-regulated in inflamed tissues [14] and is involved in the development of hyperalgesia in inflammatory and neuropathic pain models [15]. Knockout mice, providing another tool for the identification of the specific role of each isoform in different pain models, confirm the role of nNOS and iNOS isoforms [16-18]. However, studies performed on knockout mice as well as those using different selective and non-selective NOS inhibitors point to a possible role for eNOS isoform in pain processing [16,17].

NSAIDs are among the most prescribed medications, yet their efficacy is compromised by a ceiling analgesic effect. The inhibitory effect of NSAIDs on NO production has been reported in many studies both clinically [19] and experimentally [5,20] and the involvement of the NO-cGMP pathway in the antinociceptive effects of NSAIDs has been suggested in several experimental studies [21,22].

The aim of the present study was to investigate the role of NO and NOS isoforms in acute inflammatory pain induced by a clinically well-defined model of clinical pain, third molar extraction. The effect of the NSAID ketorolac on NO production and NOS gene expression was also studied. To achieve this goal, two independent studies as well as a third pilot dose finding study were carried out: (1) a microdialysis study to estimate changes in NO levels at the site of tissue injury and to correlate these levels to pain intensity reported at the same time points; (2) a gene expression study to determine change in gene expression of the three NOS isoforms at the site of tissue injury, and finally (3) the glyceryl trinitrate glyceryl trinitrate study, which aimed at finding an efficacious dose to study the effect of the NO donor glyceryl trinitrate on pain intensity following tissue injury.

## Results

### I. Association between NO level at the site of tissue injury and pain intensity

#### A. Pain intensity following tissue injury

**Figure 1:**
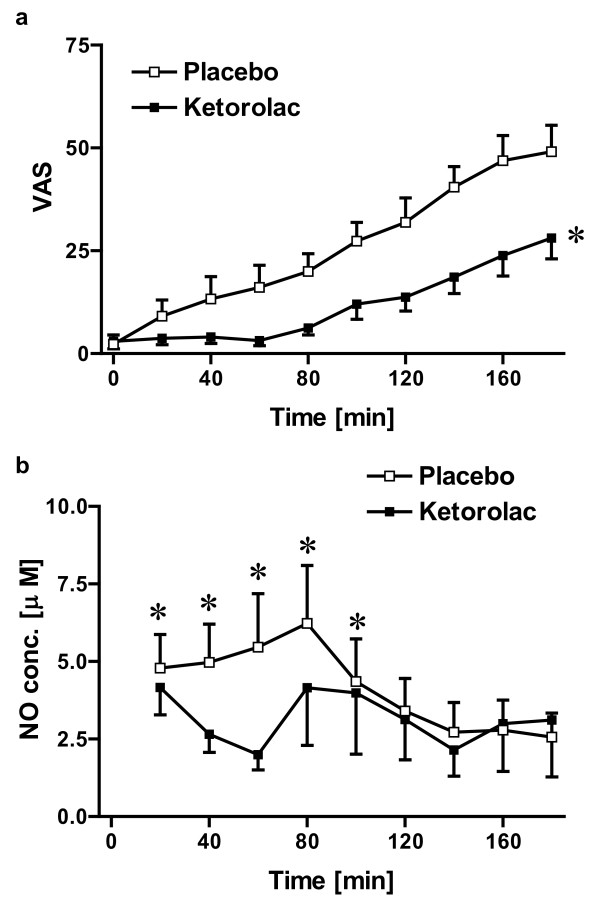
**Microdialysis Study**. **(a) **Effect of tissue injury and ketorolac treatment on pain intensity following third molar tooth extraction (p = 0.018; repeated measure two way ANOVA, n = 24 & 20 for placebo and ketorolac treatment groups, respectively). **(b) **Effect of tissue injury and ketorolac treatment on NO production at the site of oral surgery. Data are presented as mean ± SEM; n = 19 & 10 for placebo and ketorolac treatment groups, respectively; *indicates the first five time points were significantly different from the last four time points, p < 0.0001; contrast analysis under the mixed linear model. Raw data were not normally distributed so were transformed to ln(VAS+1) and statistical analysis were conducted on transformed data.

Pain intensity as evaluated by visual analogue scale (VAS) showed a continuous increase over the 3 hours observation period (Figure 1a). The repeated measure two way ANOVA model, controlling for time and the random effects of patients, detected a significant difference in pain intensity between placebo and ketorolac treated patients (p = 0.018, n = 24 & 20 for placebo and ketorolac, respectively).

#### B. NO levels at the site of tissue injury

NO levels at the surgical site were evaluated every 20 min following tissue injury using microdialysis collection. Patients that had more than one sample missing from the set of 9 serial samples due to failure of microdialysis probe, blood contamination of the sample or a low sample volume that did not allow analysis of replicates were excluded from this analysis. As seen in Figure 1b, NO levels gradually increased over the first 80 min in the placebo-treated group before declining gradually over the last 100 min of the observation period, reaching its lowest level by the end of the 3 hours collecting period. Contrast analysis under the linear mixed effects model controlling for time shows that NO levels over the first 100 min were significantly higher than those over the last 80 min (p < 0.0001) in the placebo treated group. NO level in microdialysate in subjects administered ketorolac before surgery gradually decreased over the first 60 min but was similar to placebo over the 100-180 min observations. Although, there was no significant change in the NO levels over time in the ketorolac group, the difference between the two treatment groups was not statistically significant (p = 0.48).

#### C. Correlation between pain intensity and NO levels

Because both pain intensity and NO levels were reported repeatedly in the same patients, simple correlation analysis would have ignored this fact and would have tested the correlation considering each time point as a different patient. Further, it would have ignored the difference in the time of collection or reporting of pain. Therefore linear mixed effects model was fitted to overcome both problems and to further test interaction of treatment and NO levels on pain intensity.

Only pain intensity scores for patients with successful microdialysis sampling (n = 19 & 10 for placebo and ketorolac, respectively) were included in the analysis. Under the linear mixed model with covariates time, treatment, NO, and the interaction of treatment and NO, NO was found to be negatively correlated to pain scores (regression coefficient = -0.6782, p = 0.0014, Table 1).

**Table 1:** Mixed linear model analysis solution for fixed effects on pain intensity

Effect	TTT	Estimate	Error	DF	t Value	Pr > |t|
Intercept		3.5634	0.4854	25	7.34	< .0001
T1		-2.3715	0.2329	201	-10.18	< .0001
T2		-2.3553	0.2252	201	-10.46	< .0001
T3		-1.9908	0.2243	201	-8.88	< .0001
T4		-1.3578	0.2276	201	-5.97	< .0001
T5		-1.0752	0.2217	201	-4.85	< .0001
T6		-0.6795	0.2174	201	-3.12	0.0020
T7		-0.2980	0.2161	201	-1.38	0.1696
T8		-0.1076	0.2162	201	-0.50	0.6193
T9		0				
Treatment	Placebo	0				
Treatment	Ketorolac	0.3034	0.5885	201	-0.52	0.6067
NO		-0.6782	0.2099	201	-3.23	0.0014
NO * treatment	Placebo	0				
NO * treatment	Ketorolac	-0.8911	0.2632	201	3.39	0.0009

* Data analyzed included patients with successful microdialysis, only.

### II. Effect of the NO donor glyceryl trinitrate on pain intensity

**Figure 2:**
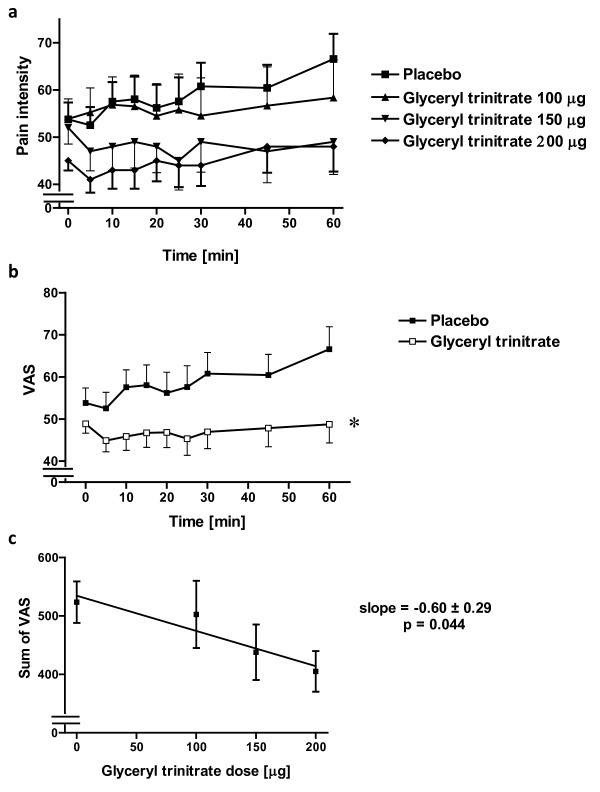
**Glyceryl trinitrate Study**. **(a) **Effect of glyceryl trinitrate (100, 150 or 200 μg) on pain intensity when infused into the surgical site of third molar tooth extraction over 20 min (n = 18-20 per group). **(b) **Effect of glyceryl trinitrate (150 or 200 μg) on pain intensity when infused into the surgical site of third molar tooth extraction over 20 min n = 20 & 37 for the placebo and glyceryl trinitrate groups respectively; p = 0.038; repeated measure 2 way ANOVA. **(c) **Dose response curve of glyceryl trinitrate's effect on pain intensity when infused into the surgical site of third molar extraction. Linear regression shows a slope significantly different from zero (n = 18-20 per group).

This study was a pilot dose finding study that aimed at finding the appropriate dose and sample size to further confirm the analgesic role of NO by administering the NO donor glyceryl trinitrate following tissue injury. Placebo or glyceryl trinitrate was infused directly into the site of surgery using PE 50 tubing. Three doses of glyceryl trinitrate were tried, none of which produced a significant analgesic effect (Figure 2a).

However, when two groups (150 and 200 μg) were pooled, there was a significant difference between the placebo and glyceryl trinitrate treated patients over the 1 hour observation period (p = 0.038, repeated measure two way ANOVA; n = 20 & 37 for placebo and glyceryl trinitrate groups, respectively; Figure 2b). Further, when the sum of pain intensity reported by each patient over the one hour observation period were plotted against the dose of glyceryl trinitrate, considering placebo as zero μg, this resulted in a linear dose response that significantly deviated from zero (p = 0.044) and that showed a negative slope of -0.60 ± 0.29; linear regression; Figure 2c).

Power analysis of the data, showed a required sample size of 47 patients per group in order to achieve an 80% power to show a difference between the sum of VAS over one hour observation period of the placebo and glyceryl trinitrate groups. Given the small effect size, the large sample size required and the increased rate of headache with the use of these doses of glyceryl trinitrate, a larger study was not conducted for ethical consideration.

### III. Effect of tissue injury and ketorolac treatment on the gene expression of NOS isoforms

#### A. Change in gene expression

**Figure 3:**
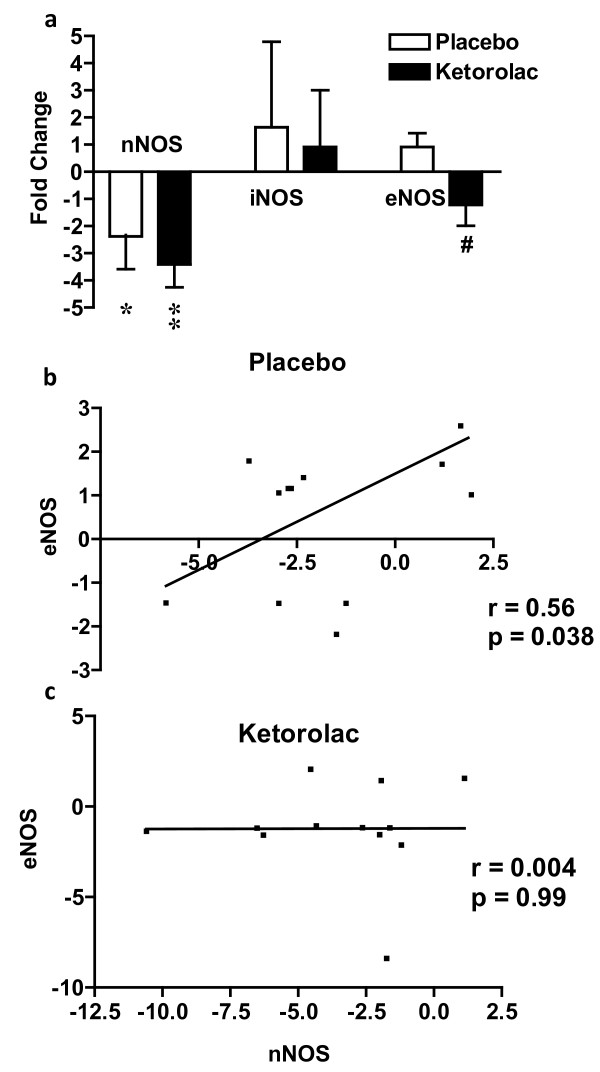
**Gene expression study**. **(a) **Tissue injury resulted in the down-regulation of gene expression of *nNOS *2.4-fold in the placebo group and 3.4-fold in the ketorolac group. *eNOS *was insignificantly up-regulated in the placebo group, but down-regulated in the ketorolac group, with significant difference between the two treatment groups. The changes in gene expression following tissue injury were assessed by qRT-PCR. The gene expression level is expressed as fold change relative to pre-surgery level. *indicates p = 0.02; **indicates p = 0.0003 compared to pre-surgery group (paired t-test); #indicates p = 0.026 compared to the placebo group (independent-sample t test). **(b & c) **The relative changes in gene expression (RQ) from qRT-PCR of *eNOS *and *nNOS *were correlated in the placebo group but not in the ketorolac treated group. The association was examined using Pearson correlation at 3 hours post-surgery.

We further studied the role of NO on acute inflammatory pain by measuring the change in gene expression of the 3 NOS isoforms using qRT-PCR. *nNOS *was significantly down-regulated in both the placebo group (2.4 fold; p = 0.02; paired t-test) and the ketorolac treatment group (3.4 fold; p = 0.0003; paired t-test). However, there was no significant difference between the two treatment groups (Figure 3a).

On the other hand, *iNOS *did not show any significant change compared to the pre-surgery level neither in the placebo group (p = 0.7) nor in the ketorolac treatment group (p = 0.98).

As for *eNOS *gene expression levels, there was a small insignificant change in both treatment groups when compared to the pre-surgery biopsies (p = 0.17 and 0.23 for the placebo and ketorolac treatment groups, respectively). However, these small changes being in opposite directions resulted in a significant difference between the two treatment groups (p = 0.026, independent two-sample t-test).

#### B. Correlation between the gene expression of NOS isoforms

The correlation between the changes in gene expression of the three NOS isoforms following tissue injury was examined using Pearson's correlation coefficients (Fig. 3b&3c). The gene expression of *nNOS *and *eNOS *were significantly correlated in the placebo group (r = 0.56; p = 0.038; n = 14; Fig. 3b), but not in the ketorolac treatment group (r = 0.004; p = 0.99; n = 14; Fig. 3c). There was no significant correlation between the changes in gene expression of *iNOS *and either of the two other isoforms (data not shown).

## Discussion

As mentioned earlier, NO has been mostly considered as a pain mediator. However, we show here in a clinical study that NO is not only devoid of this pronociceptive property in the periphery, at this early stage of acute inflammatory pain, but it is also negatively correlated to pain.

The unresolved role of NO in nociception at the periphery [8,9] has been attributed in animal studies to several factors, including the level of NO. Low levels of NO at the site of injury show antinociceptive effects, whereas higher concentrations have pronociceptive effects. In a rat model of incisional pain, the NO donor, S-nitroso-N-acetylpenicillamine (SNAP) applied inside the surgical wound, reduced the incision allodynia at concentrations between 1-10%. On the other hand, higher concentration (30%) intensified the allodynia [23]. The same finding was seen in the formalin test [24].

Baseline level of neuronal excitability is also thought to affect the role of NO in nociception [25]. Electrophysiological recordings of dural nociceptive afferents showed that neurons with higher mechanical activation thresholds were activated by NO donors, while neurons with lower mechanical activation thresholds were inhibited by NO donors [25]. Further, in PGE_2 _induced pain, the NO donor SIN-1 induced a dose dependent hypernociception when injected intradermally, while inducing an antinociceptive effect when injected subcutaneously, which suggests that different subsets of primary nociceptive neurons may respond differently to NO [26].

The NOS isoform expressed at the site of injury might also contribute to identifying the role of NO in nociception. The role of different NOS isoforms in pain processing is still subject to debate. It is known that nNOS and eNOS, though found primarily within the nervous system and endothelial tissue respectively, are also found in other tissues, whereas iNOS is expressed in various cell types, such as macrophages and neutrophils [6]. In the present study, the three *NOS *isoforms were expressed in the gingival mucosa, following tissue injury. While both nNOS [16,17,27] and iNOS [27,28] are frequently suggested to play an important pronociceptive role in several pain models, eNOS is not often studied. However, it was reported that eNOS was the only isoform involved in both phases of the rat formalin test [17] and eNOS^-/- ^mice showed a more rapid recovery from thermal hyperalgesia compared to wild type mice after intraplantar injection of complete Freund's adjuvant [16]. Interpretation of findings in NOS^-/- ^mice is compromised by the compensation of other isoforms for the deficient isoform [16,29,30], at least at the spinal level. The analgesic role of NO at the periphery may be explained by the fact that NO generated by eNOS may modulate leucocyte adherence, which contributes to inflammation [31] and pain [32]. NO is also responsible for vasodilatation with subsequent increased blood supply to the injured region and ultimately increased clearance of local inflammatory mediators [33], another possible mechanism for the analgesic effect of NO at the periphery.

The down-regulation of nNOS in both treatment groups seen here is an interesting finding that may reflect a feedback effect to the higher NO levels at earlier time points. However, the present data cannot indicate such a conclusion. Since the increase in NO production seen in the microdialysis samples occurred during the first 80 min, this may explain the minimal change noticed at the gene expression level of NOS isoforms at 3 hours. Therefore, further investigation to study the effect of tissue injury on NOS isoforms at earlier and latter time points is warranted. However, the gene expression results suggest a relatively low level of NO at the site of injury, given the down regulation of nNOS and insignificant change of the other two isoforms.

**Figure 4:**
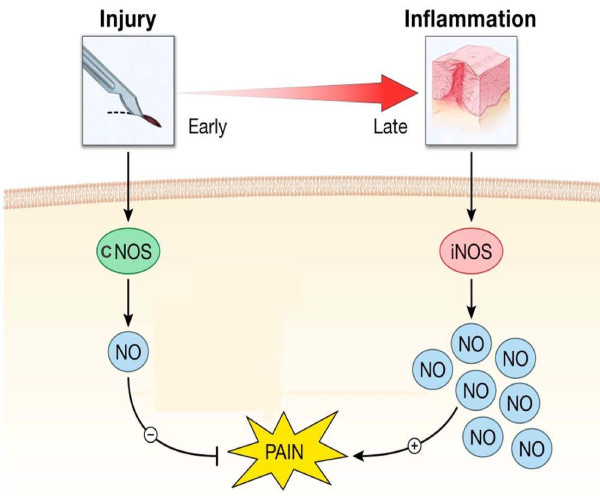
**Tissue injury increases NO production possibly via eNOS or nNOS in the early phase following surgery**. This causes a small increase in NO level that lead to an analgesic effect. However, in the late phase iNOS isoform is up-regulated, producing a marked increase in NO level that would have a hyperalgesic effect. cNOS, constitutive NOS.

Taking into account the negative correlation between NO levels and pain intensity reported here, it is plausible that at lower levels, NO released at the periphery plays an analgesic role in this clinical model of inflammatory pain (Fig. 4).

Another interesting finding in the present study is the correlation between *nNOS *and *eNOS *expression in the placebo, but not in the ketorolac treatment group, which suggests an inhibitory effect of ketorolac on *eNOS *gene expression. To our knowledge, the association between the expression of these two isoforms has not been reported earlier. It is known that both nNOS and eNOS are constitutive isoforms of NOS that are calcium-dependent [2]. We have recently reported the upregulation of kinin B_1 _and B_2 _receptors at the same pain model and time frame [34], both of which cause elevated free intracellular calcium upon activation [35] that may contribute to the activation of both NOS isoforms. The down regulation of *eNOS *by NSAIDs, on the other hand, is in line with the suppression of eNOS expression by high-dose aspirin or meloxicam, in retinas of diabetic rats [36].

The negative correlation between NO levels and pain intensity that we show here, is in line with the analgesic effect of topical glyceryl trinitrate that was reported earlier in several tendinopathies [11,33,37,38]. Unfortunately, we could not show the same analgesic effect of glyceryl trinitrate in the present study as the infusion of a rather small dose of glyceryl trinitrate (150 or 200 μg) into the surgical site did not show a significant analgesic effect at the sample size used. However, combining both groups resulted in a small analgesic effect. It was not possible to further increase the dose of glyceryl trinitrate to examine a dose dependent nature of NO effect on pain, nor to extend the study due to ethical concerns, given the small analgesic effect and the associated increased rate of headache with glyceryl trinitrate infusion.

It is not clear, why infusion of glyceryl trinitrate in the present study did not achieve a clear analgesic effect, in contrast to the earlier studies [11,33,37,38]. However, it is possible that to reach a detectable analgesic effect, longer exposure to NO is required, given in our study glyceryl trinitrate was infused for 20 min only, while in earlier studies, patients received transdermal glyceryl trinitrate patch for at least three days and up to 24 weeks.

There is a great body of evidence that supports an inhibitory role of NSAIDs on NO production both in clinical and experimental settings [19,39-41]. However, some studies suggest a stimulatory role e.g. [42,43] and others did not show any effect [44]. This variability might be due to different experimental settings, including selectivity of NSAID, concentration used, stimulants and cell type, which may reflect on the NOS isoform expressed. Nevertheless, most of the studies showing an inhibitory effect of NSAIDs on NO production also show downregulation of iNOS expression. Since in the present study iNOS isoform was not up-regulated, 3 hours following tissue injury, this may explain the failure of ketorolac to decrease NO production at this early time point. Even though ketorolac down-regulated eNOS expression, this did affect NO levels, possibly because eNOS generate small amounts of NO, compared to iNOS [2], which makes the effect of ketorolac on NO production rather small.

We therefore suggest that at this early phase of inflammatory pain, NO plays an analgesic role in the periphery. Further studies are needed to identify the contribution of NOS isoforms to this analgesic effect.

## Methods

### Subjects and procedures

The present work shows the results of two independent studies: a microdialysis study, and a gene expression study. A third, dose finding, pilot study was also conducted, glyceryl trinitrate intervention study. Participants in these three independent studies were healthy volunteers (n = 147) aged between 16 and 36 years, who required extraction of impacted third molars. Demographic data of participants in each study are shown in Table 2, 28 other subjects, who did not develop moderate pain following surgery, were excluded from the glyceryl trinitrate study. Protocols for the three studies were approved by the Institutional Review Board of NIDCR/NIH. Written informed consent was obtained from all participants before treatment. Pregnant or lactating females or patients with the presence of clinical signs of infection or inflammation at the extraction sites were not included in the study. All subjects received intravenous midazolam (4.1 ± 1 mg) and local anesthesia by 2% lidocaine (151.3 ± 25.8 mg) with epinephrine 1:100,000 prior to surgery. Following ethical regulations, a rescue analgesic drug was provided upon patient's request in both the microdialysis and gene expression studies. The observation period in case of the glyceryl trinitrate study was one hour, so rescue medicine was not permitted during this hour.

**Table 2:** Demographic data of participants

Protocol	Microdialysis				Glyceryl trinitrate				Gene Expression	
	All subjects		Successful sampling							
**Treatment**	**Placebo**	**Ketorolac**	**Placebo**	**Ketorolac**	**Placebo**	**100 μg**	**150 μg**	**200 μg**	**Placebo**	**Ketorolac**

**Number**	24	20	19	10	20	18	19	18	15	13

**Age**	20.4 ± 3.9	19.0 ± 2.4	19.9 ± 3.3	20.0 ± 2.7	22.9 ± 5.4	20.1 ± 3.6	22.0 ± 4.9	22.4 ± 5.4	19.3 ± 3.5	18.9 ± 2.8

**Gender M/F**	7/17	5/15	3/15	1/8	15/5	12/6	9/10	9/9	8/7	8/5

**Race**										

**White**	18	14	12	6	15	12	13	14	13	10

**African**	3	3	3	2	1	4	2	2	--	2

**Others**	3	3	3	1	4	2	4	2	2	1

Difficulty*	3.5 ± 0.6	3.5 ± .05	3.5 ± 0.6	3.5 ± 0.5	2.95 ± 0.8	3.2 ± 0.9	3.4 ± 0.8	3.3 ± 0.7	7.3 ± 0.9	7.2 ± 0.9

**Rescue Medicine**	14 (58%)	6 (30%)	11 (61%)	3 (33%)	N/A	N/A	N/A	N/A	9 (60%)	5 (38%)

Data presented as mean ± S.D.

* Extraction difficulty is the sum calculated by assigning a score of (2) for soft tissue impactions, (3) for partial bony impactions and (4) for full bony impactions.

### Microdialysis procedure

Following satisfactory local anesthesia, subjects randomly received either ketorolac (30 mg) or placebo intravenously immediately before surgery. Two mandibular third molars were extracted and a surgical difficulty score was assigned for each tooth. After extraction, a microdialysis probe (CMA/20 Microdialysis Probe; CMA/Microdialysis, North Chelmsford, MA) was placed along the buccal aspect of the mandible, beneath the mucogingival flap elevated for the surgical procedure [45,46]. The probe fiber consists of a 10-mm flexible, nonmetallic, semipermeable dialysis membrane with a molecular cutoff ranging from 3000 to 20,000 Da. The probes were secured to an adjacent tooth with silk suture and the flap closed using 3-0 chromic gut suture. Sterile lactated Ringer's solution was pumped at 10 μL/min and samples collected at 20-min intervals after the completion of surgery. The samples were immediately placed on dry ice after each collection period. Subjects remained under observation for the first 3 hours after surgery to evaluate pain and adverse events and for collection of samples by microdialysis. Patients rated their pain intensity every 20 min for the first 3 h post-operatively using 100-mm visual analog scale (VAS). At the conclusion of the observation period, the microdialysis probes were removed and the samples were stored at -70°C.

### Glyceryl trinitrate administration

Only patients who developed moderate pain (≥ 35 on the VAS) following surgery and disappearance of local anesthetic effect were included in this protocol. A PE50 tubing was placed under the flap elevated for tooth extraction. Three different doses of glyceryl trinitrate (100 μg, 150 μg or 200 μg) prepared in a final volume of 1 ml or a placebo were delivered over 20 min to the surgical site. These doses were selected based on the analgesic effect of transdermal glyceryl trinitrate in shoulder pain syndrome due to supraspinatus tendinitis [11]. Since this was a pilot study, with no previous experience of the possible effect size, there was no sample size calculation prior to conducting this study. The number of patients enrolled was based on previous experience with the oral surgery model. Pain was evaluated using 100 mm VAS for 1 hour following drug delivery.

### Gene expression analysis by qRT-PCR

In this protocol, two impacted third molars were extracted. A 3 mm punch biopsy was taken from the oral mucosa overlying one impacted third molar immediately prior to surgery and a second biopsy was taken from the opposite surgical site 3 hours post-surgery. All biopsies were immediately frozen in liquid nitrogen and stored at -70°C until ready for RNA extraction. Patients received a placebo or ketorolac 30 minutes prior to surgery.

Oral mucosal biopsies (*n *= 64) were used to detect gene expression using ABI Prism 7900 HT Sequence Detection System (Applied Biosystems, Foster City, CA) as described previously [45,47,48]. All reagents were purchased from Applied Biosystems and 2 μg of DNase-treated RNA was used to synthesize cDNA using random primers from the High-Capacity cDNA Archive Kit according to the manufacturer's instruction. Polymerase chain reaction was performed with cDNA template using the PCR Master Mix with AmpErase UNG. Sequence-specific primers and TaqMan MGB probes were purchased from Assays-on-Demand Gene expression product. Quantification of gene expression was performed in a 20-μL reaction (384-well plate) and each sample was run in triplicate. The housekeeping gene 18S rRNA was used as endogenous control and negative controls were processed under the same conditions without a cDNA template. Data acquisition was conducted using User Bulletin #2 software (v1.6, Applied Biosystems). The threshold cycle (Ct) of 18 rRNA was used to normalize target gene expression (ΔCt) to correct for experimental variations. The relative change in gene expression (ΔΔCt) was used for comparison of the gene expression in post-surgery tissue versus that in pre-surgery tissue.

### Measurement of NO degradation products

NO has a very short half-life and is quickly degraded in-vivo to its stable end-products nitrite and nitrate. Nitrite and nitrate (NOx) as an index of total NO production were measured in microdialysis samples using a commercially available kit (Nitrate/Nitrite Fluorometric Assay Kit, Cayman Chemical Company, Ann Arbor, MI), following the manufacturer's instructions. Samples containing blood were excluded as blood interfers with accurate evaluation of nitrite and nitrate levels using fluorometry. The samples were run in triplicate and fluorescence was read using a Victor^3 ^Perkins Elmer spectrofluorometer (excitation = 370 nm; emission = 420 nm).

Fluorometric assay for NOx measurement is more sensitive than the conventional colourimetric assays based on Griess reaction and both methods were found to produce essentially the same results [49].

### Statistical analysis

Statistical analysis was carried out using SAS (v. 9.1, SAS Institute Inc., Cary, NC) or SPSS (v. 16.0, SPSS, Chicago, IL). Demographic data, such as age and gender, were summarized for different treatment groups. Mean and SEM of NO and VAS were plotted for ketorolac and NTG treatment at 9 time points. Glyceryl trinitrate dose response was evaluated using linear regression. Comparisons between effects of ketorolac or glyceryl trinitrate treatment and placebo on VAS were performed using repeated measure two-way ANOVA. Linear mixed effects models were fitted to investigate the effect of time (at 9 levels), treatment, NO and the interaction effect between treatment and NO on VAS for the microdialysis study. Patient ID was assumed to be a random effect and each patient had a random intercept in all the linear mixed models since the samples were pulled out from a large population. The effect of treatment on NO production was also examined using linear mixed effect models. Data that did not follow normal distribution was transformed to ln(x+1). The relative change in gene expression was assessed by paired t-test, and the comparison between treatment groups was assessed by independent two-sample t test and the association among these gene expressed was examined by Pearson's correlation coefficients. Results were considered significant at α = 0.05.

## Conflict of interests

The authors declare that they have no competing interests.

## Authors' contributions

MH contributed to the laboratory experimental design, conducted NOx measurments, qRT-PCR experiments, data analysis and manuscript writing. XMW participated in the laboratory experimental design, conducted RNA extraction, qRT-PCR experiments and data acquisition, and editing the manuscript. TW contributed to statistical data analysis and editing the manuscript. JSB, JSR and RAD participated in the patient enrollment, surgical procedures, patient care and specimen collection. RAD was entirely responsible for the overall study design, overseeing data collection, analysis and interpretation as well as manuscript version. All authors have read and approved the final manuscript.

## References

[B1] Schuman EM Madison DV Nitric oxide and synaptic function Annual Review of Neuroscience 1994 17 153 183 10.1146/annurev.ne.17.030194.001101 7516125

[B2] Miclescu A Gordh T Nitric oxide and pain: 'Something old, something new' Acta Anaesthesiologica Scandinavica 2009 53 1107 1120 10.1111/j.1399-6576.2009.02054.x 19702699

[B3] McMahon SB Bennett DLH Bevan S McMahon SB, Koltzenburg M Inflammatory mediators and modulators of pain Wall and Melzack's Textbook of Pain 2006 Edinburgh: Elsevier Churchill Livingstone 49 72

[B4] Vetter G Geisslinger G Tegeder I Release of glutamate, nitric oxide and prostaglandin E2 and metabolic activity in the spinal cord of rats following peripheral nociceptive stimulation Pain 2001 92 213 218 10.1016/S0304-3959(01)00258-5 11323142

[B5] Gühring H Hamza M Sergejeva M Ates M Kotalla CE Ledent C Brune K A role for endocannabinoids in indomethacin-induced spinal antinociception Eur J Pharmacol 2002 454 153 163 10.1016/S0014-2999(02)02485-8 12421642

[B6] Osborne MG Coderre TJ Effects of intrathecal administration of nitric oxide synthase inhibitors on carrageenan-induced thermal hyperalgesia Br J Pharmacol 1999 126 1840 1846 10.1038/sj.bjp.0702508 10372828 PMC1565961

[B7] Schmidtko A Tegeder I Geisslinger G No NO, no pain? The role of nitric oxide and cGMP in spinal pain processing Trends Neurosci 2009 32 339 346 10.1016/j.tins.2009.01.010 19414201

[B8] Durate ID Lorenzetti BB Ferreira SH Peripheral analgesia and activation of the nitric oxide-cyclic GMP pathway Eur J Pharmacol 1990 186 289 293 10.1016/0014-2999(90)90446-D 1981187

[B9] Aley KO McCarter G Levine JD Nitric Oxide Signaling in Pain and Nociceptor Sensitization in the Rat J Neurosci 1998 18 7008 7014 9712669 10.1523/JNEUROSCI.18-17-07008.1998 PMC6792985

[B10] Holthusen H Arndt JO Nitric oxide evokes pain in humans on intracutaneous injection Neurosci Lett 1994 165 71 74 10.1016/0304-3940(94)90712-9 8015741

[B11] Berrazueta JR Losada A Poveda J Ochoteco A Riestra A Salas E Amado JA Successful treatment of shoulder pain syndrome due to supraspinatus tendinitis with transdermal nitroglycerin. A double blind study Pain 1996 66 63 67 10.1016/0304-3959(96)03021-7 8857632

[B12] Brisby H Ashley H Diwan AD In vivo measurement of facet joint nitric oxide in patients with chronic low back pain Spine 2007 32 1488 1492 10.1097/BRS.0b013e318067dc97 17572616

[B13] Bica BE Gomes NM Fernandes PD Luiz RR Koatz VL Nitric oxide levels and the severity of juvenile idiopathic arthritis Rheumatol Int 2007 27 819 825 10.1007/s00296-007-0321-x 17287934

[B14] Gühring H Tegeder I Lötsch J Pahl A Werner U Reeh PW Rehse K Brune K Geisslinger G Role of nitric oxide in zymosan induced paw inflammation and thermal hyperalgesia Inflammation Research 2001 50 83 88 10.1007/s000110050728 11289658

[B15] Jorge De A Nick MC Sue DC Philip C Iain C Richard GK GW274150, a novel and highly selective inhibitor of the inducible isoform of nitric oxide synthase (iNOS), shows analgesic effects in rat models of inflammatory and neuropathic pain Pain 2006 120 170 181 10.1016/j.pain.2005.10.028 16360270

[B16] Boettger MK Üceyler N Zelenka M Schmitt A Reif A Chen Y Sommer C Differences in inflammatory pain in nNOS-, iNOS- and eNOS-deficient mice European Journal of Pain 2007 11 810 818 10.1016/j.ejpain.2006.12.008 17395508

[B17] Doursout M-F Liang Y Chelly JE NOS inhibitors exhibit antinociceptive properties in the rat formalin test Can J Anesth 2003 50 909 916 10.1007/BF03018738 14617588

[B18] Gühring H Gorig M Ates M Coste O Zeilhofer HU Pahl A Rehse K Brune K Suppressed Injury-Induced Rise in Spinal Prostaglandin E2 Production and Reduced Early Thermal Hyperalgesia in iNOS-Deficient Mice J Neurosci 2000 20 6714 6720 10964977 10.1523/JNEUROSCI.20-17-06714.2000 PMC6772960

[B19] Vandivier RW Eidsath A Banks SM Preas HL Leighton SB Godin PJ Suffredini AF Danner RL Down-regulation of nitric oxide production by ibuprofen in human volunteers J Pharmacol Exp Ther 1999 289 1398 1403 10336532

[B20] Ryu YS Lee JH Seok JH Hong JH Lee YS Lim JH Kim YM Hur GM Acetaminophen inhibits iNOS gene expression in RAW 264.7 macrophages: differential regulation of NF-kappaB by acetaminophen and salicylates Biochem Biophys Res Commun 2000 272 758 764 10.1006/bbrc.2000.2863 10860828

[B21] Granados-Soto V Flores-Murrieta FJ Castaneda-Hernandez G Lopez-Munoz FJ Evidence for the involvement of nitric oxide in the antinociceptive effect of ketorolac Eur J Pharmacol 1995 277 281 284 10.1016/0014-2999(95)00123-3 7493621

[B22] Ventura-Martinez R Deciga-Campos M Diaz-Reval MI Gonzalez-Trujano ME Lopez-Munoz FJ Peripheral involvement of the nitric oxide-cGMP pathway in the indomethacin-induced antinociception in rat Eur J Pharmacol 2004 503 43 48 10.1016/j.ejphar.2004.09.018 15496294

[B23] Prado WA Schiavon VF Cunha FQ Dual effect of local application of nitric oxide donors in a model of incision pain in rats Eur J Pharmacol 2002 441 57 65 10.1016/S0014-2999(02)01413-9 12007920

[B24] Kawabata A Manabe S Manabe Y Takagi H Effect of topical administration of L-arginine on formalin-induced nociception in the mouse: a dual role of peripherally formed NO in pain modulation Br J Pharmacol 1994 112 547 550 7521259 10.1111/j.1476-5381.1994.tb13108.x PMC1910365

[B25] Levy D Strassman AM Modulation of dural nociceptor mechanosensitivity by the nitric oxide-cyclic GMP signaling cascade J Neurophysiol 2004 92 766 772 10.1152/jn.00058.2004 15056690

[B26] Vivancos GG Parada CA Ferreira SH Opposite nociceptive effects of the arginine/NO/cGMP pathway stimulation in dermal and subcutaneous tissues British Journal of Pharmacology 2003 138 1351 1357 10.1038/sj.bjp.0705181 12711636 PMC1573782

[B27] Tanabe M Nagatani Y Saitoh K Takasu K Ono H Pharmacological assessments of nitric oxide synthase isoforms and downstream diversity of NO signaling in the maintenance of thermal and mechanical hypersensitivity after peripheral nerve injury in mice Neuropharmacology 2009 56 702 708 10.1016/j.neuropharm.2008.12.003 19111753

[B28] Celerier E Gonzalez JR Maldonado R Cabanero D Puig MM Opioid-induced hyperalgesia in a murine model of postoperative pain: role of nitric oxide generated from the inducible nitric oxide synthase Anesthesiology 2006 104 546 555 10.1097/00000542-200603000-00023 16508403

[B29] Tao F Tao YX Mao P Zhao C Li D Liaw WJ Raja SN Johns RA Intact carrageenan-induced thermal hyperalgesia in mice lacking inducible nitric oxide synthase Neuroscience 2003 120 847 854 10.1016/S0306-4522(03)00362-2 12895524

[B30] Tao F Tao YX Zhao C Dore S Liaw WJ Raja SN Johns RA Differential roles of neuronal and endothelial nitric oxide synthases during carrageenan-induced inflammatory hyperalgesia Neuroscience 2004 128 421 430 10.1016/j.neuroscience.2004.06.038 15350652

[B31] Kubes P Suzuki M Granger DN Nitric oxide: an endogenous modulator of leukocyte adhesion Proceedings of the National Academy of Sciences of the United States of America 1991 88 4651 4655 10.1073/pnas.88.11.4651 1675786 PMC51723

[B32] Cunha TM Verri WA Jr Schivo IR Napimoga MH Parada CA Poole S Teixeira MM Ferreira SH Cunha FQ Crucial role of neutrophils in the development of mechanical inflammatory hypernociception J Leukoc Biol 2008 83 824 832 10.1189/jlb.0907654 18203872

[B33] Paoloni JA Appleyard RC Nelson J Murrell GA Topical nitric oxide application in the treatment of chronic extensor tendinosis at the elbow: a randomized, double-blinded, placebo-controlled clinical trial Am J Sports Med 2003 31 915 920 14623657 10.1177/03635465030310062901

[B34] Hamza M Wang X-M Adam A Brahim J Rowan J Carmona G Dionne R Kinin B1 receptors contributes to acute pain following minor surgery in humans Molecular Pain 2010 6 12 10.1186/1744-8069-6-12 20152050 PMC2834653

[B35] Leeb-Lundberg LMF Marceau F Muller-Esterl W Pettibone DJ Zuraw BL International union of pharmacology. XLV. Classification of the kinin receptor family: from molecular mechanisms to pathophysiological consequences Pharmacol Rev 2005 57 27 77 10.1124/pr.57.1.2 15734727

[B36] Joussen AM Poulaki V Mitsiades N Kirchhof B Koizumi K Dohmen S Adamis AP Nonsteroidal anti-inflammatory drugs prevent early diabetic retinopathy via TNF-α suppression FASEB J 2002 01 0707fje 10.1096/fj.01-0707fje 11821258

[B37] Paoloni JA Appleyard RC Nelson J Murrell GA Topical glyceryl trinitrate treatment of chronic noninsertional achilles tendinopathy. A randomized, double-blind, placebo-controlled trial J Bone Joint Surg Am 2004 86-A 916 922 15118032 10.2106/00004623-200405000-00005

[B38] Paoloni JA Appleyard RC Nelson J Murrell GA Topical glyceryl trinitrate application in the treatment of chronic supraspinatus tendinopathy: a randomized, double-blinded, placebo-controlled clinical trial Am J Sports Med 2005 33 806 813 10.1177/0363546504270998 15827365

[B39] Hrabak A Vercruysse V Kahan IL Vray B Indomethacin prevents the induction of inducible nitric oxide synthase in murine peritoneal macrophages and decreases their nitric oxide production Life Sci 2001 68 1923 1930 10.1016/S0024-3205(01)00978-X 11292070

[B40] Matsuda KK Nakamura SS Matsushita TT Celecoxib inhibits nitric oxide production in chondrocytes of ligament-damaged osteoarthritic rat joints Rheumatol Int 2006 26 991 995 10.1007/s00296-006-0107-6 16437200

[B41] Sprott H Gay RE Michel BA Gay S Influence of ibuprofen-arginine on serum levels of nitric oxide metabolites in patients with chronic low back pain--a single-blind, placebo controlled pilot trial (ISRCTN18723747) J Rheumatol 2006 33 2515 2518 17013995

[B42] Paul-Clark MJ van Cao T Moradi-Bidhendi N Cooper D Gilroy DW 15-epi-lipoxin A4-mediated Induction of Nitric Oxide Explains How Aspirin Inhibits Acute Inflammation J Exp Med 2004 200 69 78 10.1084/jem.20040566 15238606 PMC2213311

[B43] Shimpo M Ikeda U Maeda Y Ohya K-i Murakami Y Shimada K Effects of Aspirin-Like Drugs on Nitric Oxide Synthesis in Rat Vascular Smooth Muscle Cells Hypertension 2000 35 1085 1091 10818069 10.1161/01.hyp.35.5.1085

[B44] Amin AAR Vyas PP Attur MM Leszczynska-Piziak JJ Patel IIR Weissmann GG Abramson SSB The mode of action of aspirin-like drugs: effect on inducible nitric oxide synthase Proc Natl Acad Sci USA 1995 92 7926 7930 10.1073/pnas.92.17.7926 7544010 PMC41259

[B45] Gordon SM Chuang BP Wang XM Hamza MA Rowan JS Brahim JS Dionne RA The differential effects of bupivacaine and lidocaine on prostaglandin E2 release, cyclooxygenase gene expression and pain in a clinical pain model Anesth Analg 2008 106 321 327 10.1213/01.ane.0000296474.79437.23 18165598

[B46] Lee Y-S Kim H Brahim JS Rowan J Lee G Dionne RA Acetaminophen selectively suppresses peripheral prostaglandin E2 release and increases COX-2 gene expression in a clinical model of acute inflammation Pain 2007 129 279 286 10.1016/j.pain.2006.10.020 17175104

[B47] Wang X-M Wu T-X Hamza M Ramsay ES Wahl SM Dionne RA Rofecoxib modulates multiple gene expression pathways in a clinical model of acute inflammatory pain Pain 2007 128 136 147 10.1016/j.pain.2006.09.011 17070997 PMC1894940

[B48] Wang XM Hamza M Gordon SM Wahl SM Dionne RA COX Inhibitors downregulate PDE4D expression in a clinical model of inflammatory pain Clin Pharmacol Ther 2008 84 39 42 10.1038/sj.clpt.6100501 18288087

[B49] Flam BR Eichler DC Solomonson LP Endothelial nitric oxide production is tightly coupled to the citrulline-NO cycle Nitric Oxide 2007 17 115 121 10.1016/j.niox.2007.07.001 17869551

